# Discrepancies between parent-reported and clinician-rated autism severity: roles of sex and age

**DOI:** 10.3389/fpsyt.2026.1823090

**Published:** 2026-08-17

**Authors:** Rio Ishida, Kazuhiko Yamamuro, Natsuko Kashida, Tsutomu Takeda, Michihiro Toritsuka, Takashi Okada, Manabu Makinodan

**Affiliations:** 1 Department of Neuropsychiatry, Kumamoto University, Kumamoto, Japan; 2 Center for Health Control, Nara Medical University, Kashihara, Japan; 3 Department of Psychiatry, Nara Medical University School of Medicine, Kashihara, Japan

**Keywords:** ADOS-2, AQ-J, autism spectrum disorder, discrepancy, sex differences, SRS-2

## Abstract

**Introduction:**

Assessment of autism spectrum disorder (ASD) relies on both parent-reported questionnaires and clinician-administered observational instruments. Although discrepancies between informants are common, their implications for autism severity evaluation remain unclear. This study examined informant discrepancies across parent-reported and clinician-rated measures, focusing on sex- and age-related effects.

**Methods:**

Children and adolescents with ASD and typically developing (TD) controls were assessed using parent-reported measures, including the Social Responsiveness Scale, Second Edition (SRS-2), and the Japanese version of the Autism-Spectrum Quotient (AQ-J). Clinician-rated autism severity was assessed using the Autism Diagnostic Observation Schedule, Second Edition (ADOS-2). Two-way analyses of variance were used to examine the diagnostic and sex-related effects. Hierarchical regression analyses were used to evaluate the associations between parent-reported subscales and ADOS-2 severity within the ASD group. Spearman's rank correlations were used to assess age-related changes in discrepancy scores separately by sex.

**Results:**

Parent-reported measures robustly differentiated the ASD and TD groups but showed few sex-related differences. In contrast, the ADOS-2 total score differed significantly by sex within the ASD group, with males exhibiting higher clinician-rated autism severity; however, most domain-level scores were not significant. Hierarchical regression analyses indicated limited associations between parent-reported subscales and clinician-rated severity. Sex remained a consistent predictor of ADOS-2 total scores. Age was negatively associated with SRS-2–ADOS-2 discrepancy scores among females, suggesting reduced parent–clinician discrepancy with increasing age in girls.

**Conclusion:**

Parent-reported and clinician-rated measures captured overlapping but distinct aspects of autism-related characteristics. The ADOS-2 total score identified a sex-related difference in clinician-rated severity that was not apparent in parent-reported measures; however, these findings should be interpreted cautiously, given documented limitations of the ADOS-2 in detecting some autistic female presentations. Overall, these findings highlight the complementary contributions of parent-reported and clinician-rated assessments and underscore the importance of considering informant perspectives when interpreting autism-related characteristics across contexts.

## 1 Introduction

Autism spectrum disorder (ASD) is a neurodevelopmental condition characterized by persistent challenges in social communication and interaction along with restricted and repetitive behaviors (1). The clinical presentation of ASD varies across individuals in terms of symptom severity, cognitive ability, developmental stage, and contextual expression (2, 3). Therefore, accurate assessment relies on integrating information from multiple sources, including caregivers and trained clinicians (4). Accumulating evidence suggests the existence of a proposed female autism phenotype, characterized by stronger social motivation, greater use of camouflaging strategies, and subtler presentation of core symptoms, which may contribute to underidentification in females (5, 6).

However, discrepancies between informants are common in the assessment of child and adolescent psychopathology (7, 8). The informant discrepancy theory proposes that differences across raters do not necessarily reflect measurement error but may instead capture meaningful, context-specific behavioral variation (7, 9, 10). Parents typically observe children across naturalistic and longitudinal contexts, whereas clinicians evaluate behavior using structured, time-limited assessments. These contextual differences may lead to a systematic divergence between questionnaire-based reports and clinician-administered observational instruments (11).

The Autism Diagnostic Observation Schedule–Second Edition (ADOS-2) is a widely used, standardized clinician-administered instrument for evaluating symptom severity in ASD (12). It provides standardized observational measures of social affect and restricted/repetitive behaviors within a structured interaction paradigm. In contrast, caregiver-report measures such as the Social Responsiveness Scale–Second Edition (SRS-2) (13) and Autism-Spectrum Quotient (AQ) (14), including its Japanese version (AQ-J) (15), assess autism-related traits as they manifest in everyday social environments. These parent-reported instruments demonstrate strong psychometric properties and are sensitive to individual differences in autistic traits (13, 16). Conceptually, the ADOS-2 measures observable behavior elicited under standardized testing conditions, whereas parent-reported instruments index broader behavioral tendencies and functional impact across naturalistic everyday contexts; these represent complementary but not interchangeable constructs.

Despite their widespread use, correlations between parent-reported measures and clinician-rated severity are often modest (17, 18). Findings from meta-analyses indicate that cross-informant agreement in ASD is typically lower than within-informant agreement (8). This pattern poses important questions regarding the parameters captured by each assessment modality and the interpretation of discrepancies. Insights into informant discrepancies are particularly relevant for clinical decision-making, research design, and the interpretation of sex-related and developmental effects.

Sex-related differences in the prevalence estimates of ASD have been extensively documented (19). Notably, these differences are frequently attributed in part to diagnostic instruments that were developed and normed predominantly in male samples, which may limit their sensitivity to the autistic presentation in females (5, 20). However, whether sex differences are similarly expressed across informants remains unclear. Some studies report few sex differences in parent-reported measures but greater differences in clinician-rated assessments (21, 22). Such findings suggest that sex-related effects may interact with the assessment modality rather than reflect consistent differences in symptom severity. Clarifying whether sex differentially influences parent-reported and clinician-rated measures is therefore important for interpreting diagnostic patterns and severity ratings.

Developmental factors may also influence informant agreement. Age-related changes in social cognition, communication skills, and self-regulation may alter both behavioral expression and observer interpretation (23, 24). Parent–clinician agreement may vary across developmental stages (25, 26); however, empirical evidence remains limited. Therefore, examining age-related variation in discrepancy scores may provide insight into the developmental influences on cross-informant agreement. Importantly, sex may moderate the patterns of informant agreement across development; in females, age-related differences in camouflaging capacity have been hypothesized as one possible factor that may influence how autistic characteristics are expressed and perceived across assessment contexts, though direct empirical evidence remains limited (27).

Overall, despite the central role of parent-reported and clinician-rated measures in ASD assessment, the extent of their agreement, and whether this agreement varies according to sex and age, remains insufficiently characterized. Few studies have systematically examined diagnostic effects, sex differences, hierarchical associations, and age-related discrepancies across multiple parent-reported and clinician-rated measures within the same cohort.

Therefore, in this study, we investigated informant discrepancies in ASD by examining the association between parent-reported measures (SRS-2 and AQ-J) and clinician-rated severity (ADOS-2). Specifically, we examined diagnostic and sex-related effects across parent-reported and clinician-rated instruments, hierarchical associations between subscale scores and clinician-rated severity within the ASD group, and age-related variations in parent–clinician discrepancy. By situating the findings within an informant discrepancy framework, we sought to clarify how assessment modalities may shape the interpretation of autism-related characteristics across contexts.

## 2 Materials and methods

### 2.1 Participants

In total, 79 children and adolescents aged 9–15 years participated in this study, including 40 children with a clinical diagnosis of ASD and 39 typically developing (TD) controls. Participants in the ASD group were recruited from among patients receiving care at the Department of Psychiatry, Nara Medical University Hospital, between March 2021 and January 2024. Eligible patients were enrolled after written informed consent was obtained from their parents or legal guardians. Participants in the TD group were recruited through convenience sampling from the local community. This approach may introduce selection bias, as TD controls were not formally matched to the ASD group on characteristics beyond age and sex, which should be considered when interpreting between-group comparisons. ASD was diagnosed by at least two experienced psychiatrists or clinical psychologists, according to the criteria of the Diagnostic and Statistical Manual of Mental Disorders, 5th edition (DSM-5) (1). To enhance diagnostic accuracy, only individuals who met ASD classification criteria on the Autism Diagnostic Observation Schedule, Second Edition (ADOS-2; Modules 3–4) were included. The ADOS-2 has demonstrated good diagnostic validity in differentiating autism from other developmental and psychiatric conditions (28). A detailed flow diagram of participant enrollment and exclusion procedures is presented in **Supplementary Figure 1**.

Cognitive function was assessed using the Das–Naglieri Cognitive Assessment System (DN-CAS), a theory-driven instrument that evaluates four cognitive processing domains: planning, attention, simultaneous processing, and successive processing (29). Participants with a total DN-CAS score below 70 were excluded to minimize the influence of intellectual disability, consistent with previous studies that have used scores below 70 as an indicator of significant cognitive impairment (29). Individuals with other neurological or psychiatric disorders were also excluded from the study. It should be noted that the DN-CAS provides scores across four processing domains but does not yield a traditional Full-Scale Intelligence Quotient (FSIQ), which may limit direct comparability with studies that characterize cognitive functioning using conventional IQ-based measures.

Demographic information, including age and sex, was obtained from caregiver questionnaires and medical records. Written informed consent was obtained from all parents or legal guardians, and assent was obtained from all children when appropriate. The study protocol was approved by the Ethics Committee of Nara Medical University (approval no. 1319).

### 2.2 Social responsiveness scale–second edition

Parent-reported social responsiveness was assessed using the Social Responsiveness Scale–Second Edition (SRS-2) (13). The SRS-2 is a 65-item questionnaire designed to measure the severity of ASD-related social impairments in naturalistic social contexts. Each item is rated on a 4-point Likert scale ranging from 0 (‘not true’) to 3 (‘almost always true’). Raw scores are converted to standardized T-scores (mean = 50, SD = 10), with higher scores indicating greater social impairment. The SRS-2 has five treatment-related subscales: Social Awareness, Social Cognition, Social Communication, Social Motivation, and Restricted Interests and Repetitive Behavior. In addition, DSM-5–aligned composite indices are available, including Social Communication and Interaction (SCI) and Restricted and Repetitive Behavior (RRB). The SRS-2 has demonstrated good internal consistency, test–retest reliability, and convergent validity with clinician-rated measures of autism severity (13).

### 2.3 Autism spectrum quotient–Japanese version

The AQ-J is a 50-item questionnaire that evaluates autistic traits across five core domains: social skills, attention switching, attention to detail, communication, and imagination. Each item is rated on a 4-point Likert scale, with higher scores indicating greater levels of autistic traits. Examples include “I find it difficult to make new friends” (social skills), “I frequently get so absorbed in one thing that I lose sight of other things” (attention switching), and “I find it difficult to read between the lines in conversations” (communication). The AQ-J has been validated for use in Japanese populations and demonstrates good internal consistency (Cronbach’s α = 0.77–0.86) (30). In the present study, because participants were aged 16 years or younger, the questionnaire was completed by a parent or caregiver. Caregiver completion of the AQ-J for participants aged 16 years or younger was adopted in line with administration practices used in prior pediatric research (31).

### 2.4 Autism diagnostic observation schedule–second edition

The ADOS-2 is a semi-structured, standardized observational instrument used to assess autism-related behaviors and support diagnostic evaluations. Modules 3 and 4, which are intended for verbally fluent children, adolescents, and adults, were administered by trained clinicians in the present study. For the purposes of this study, domain scores for Reciprocal Social Interaction, Language and Communication, Social Affect, and Restricted and Repetitive Behaviors, as well as a total severity score, were extracted from the ADOS-2. The total severity score reflects the severity of autism-related behaviors observed during the standardized assessment. The ADOS-2 has demonstrated good diagnostic validity and reliability in differentiating ASD from other developmental conditions (28). It should be noted, however, that normative data for the ADOS-2 were derived predominantly from male samples, and accumulating evidence suggests that this may limit its sensitivity for detecting autism in females (5, 20).

### 2.5 Statistical analyses

All statistical analyses were performed using SPSS (version 26; IBM Corp., Armonk, NY, USA). Statistical significance was defined as a two-tailed p-value < 0.05, unless otherwise specified.

Group differences in categorical variables were examined using chi-square (χ^2^) tests, and group differences in continuous variables were examined using analysis of variance (ANOVA) or independent-samples t-tests, as appropriate. To examine the effects of diagnostic group (TD vs. ASD) and sex (male vs. female) on SRS-2 and AQ-J scores, two-way ANOVAs were conducted for total scores and subscales. The main effects of diagnosis and sex, as well as their interactions, were evaluated. To control for multiple comparisons, Benjamini-Hochberg (BH) false discovery rate (FDR) correction was applied separately to the p-values for each effect type (sex, diagnosis, and interaction) within each family of analyses (eight tests for SRS-2 measures; six tests for AQ-J measures). In **Table 1** and **Supplementary Table 1**, values that remained statistically significant after FDR correction are indicated in bold. Within the ASD group, sex differences in clinician-rated autism severity (ADOS-2 total and domain scores) were analyzed using independent-samples t-tests (statistically equivalent to one-way ANOVA for two-group comparisons).

**Table 1:** Social responsiveness scale–second edition (SRS-2) scores according to diagnostic group and sex.

	TD		ASD				
Measure	Male Mean (SD)	Female Mean (SD)	Male Mean (SD)	Female Mean (SD)	Sex (F, p, Partial η^2^)	Diagnosis (F, p, Partial η^2^)	Interaction (F, p, Partial η^2^)
Total score	48.87 (7.49)	43.31 (7.30)	72.40 (12.92)	71.05 (12.91)	*F*_1,75_ = 2.075 *p* = 0.154 Partial η^2^ = 0.027	***F*_1,75_ = 114.318 *p* = 9.750×10^-17^** **Partial η^2^ = 0.604**	*F*_1,75_ = 0.770 *p* = 0.383 Partial η^2^ = 0.010
Social awareness	49.65 (10.87)	44.56 (8.89)	62.05 (12.68)	58.25 (15.01)	*F*_1,75_ = 2.587 *p* = 0.112 Partial η^2^ = 0.033	***F*_1,75_ = 22.273 *p* = 1.074×10** ^-5^ **Partial η^2^ = 0.229**	*F*_1,75_ = 0.054 *p* = 0.816 Partial η^2^ = 0.001
Social cognition	50.00 (9.71)	41.75 (7.72)	71.85 (13.54)	70.55 (14.52)	*F*_1,75_ = 3.176 *p* = 0.079 Partial η^2^ = 0.041	***F*_1,75_ = 89.350 *p* = 2.061×10** ^-14^ **Partial η^2^ = 0.544**	*F*_1,75_ = 1.682 *p* = 0.199 Partial η^2^ = 0.022
Social communication	48.13 (7.27)	44.94 (8.16)	67.00 (10.96)	69.05 (12.36)	*F*_1,75_ = 0.065 *p* = 0.800 Partial η^2^ = 0.001	***F*_1,75_ = 91.566 *p* = 1.241×10** ^-14^ **Partial η^2^ = 0.550**	*F*_1,75_ = 1.362 *p* = 0.247 Partial η^2^ = 0.018
Social motivation	48.48 (8.47)	44.25 (6.67)	69.55 (12.07)	64.50 (13.24)	*F*_1,75_ = 3.757 *p* = 0.056 Partial η^2^ = 0.048	***F*_1,75_ = 74.523 *p* = 7.446×10** ^-13^ **Partial η^2^ = 0.529**	*F*_1,75_ = 0.029 *p* = 0.864 Partial η^2^ = 3.928**×**10^-4^
Repetitive behaviors and restricted interest	48.91 (8.31)	46.38 (6.21)	72.85 (15.66)	73.25 (15.26)	*F*_1,75_ = 0.149 *p* = 0.701 Partial η^2^ = 0.002	***F*_1,75_ = 84.118 *p* = 7.027×10** ^-14^ **Partial η^2^ = 0.498**	*F*_1,75_ = 0.281 *p* = 0.597 Partial η^2^ = 0.004
SCI	48.83 (7.50)	43.06 (7.42)	69.65 (12.51)	69.40 (12.73)	*F*_1,75_ = 1.623 *p* = 0.207 Partial η^2^ = 0.021	***F*_1,75_ = 99.791 *p* = 1.999×10** ^-15^ **Partial η^2^ = 0.571**	*F*_1,75_ = 1.364 *p* = 0.247 Partial η^2^ = 0.018
RRB	48.91 (8.31)	45.75 (7.11)	72.85 (15.66)	73.45 (14.79)	*F*_1,75_ = 0.216 *p* = 0.644 Partial η^2^ = 0.003	***F*_1,75_ = 87.542 *p* = 3.135×10** ^-14^ **Partial η^2^ = 0.539**	*F*_1,75_ = 0.465 *p* = 0.497 Partial η^2^ = 0.006

Values are presented as mean and standard deviation (SD). Social functioning and autism-related social impairments were assessed using the Social Responsiveness Scale–Second Edition (SRS-2), including the total score, five treatment subscales (Social Awareness, Social Cognition, Social Communication, Social Motivation, and Restricted Interests and Repetitive Behavior), and DSM-5–aligned indices (Social Communication and Interaction [SCI] and Restricted and Repetitive Behavior [RRB]). Two-way analysis of variance (ANOVA) was performed for each SRS-2 measure, with the diagnostic group (typically developing [TD] vs. autism spectrum disorder [ASD]) and sex (male vs. female) as between-subject factors. F values, p-values, and effect sizes are reported for the main effects of sex and diagnosis, as well as for their interaction. Effect sizes are expressed as partial eta squared (partial η^2^). Benjamini-Hochberg (BH) false discovery rate (FDR) correction was applied separately to the p-values for each effect type (sex, diagnosis, and interaction) across the eight SRS-2 measures. Bold values indicate statistical significance after FDR correction. A two-tailed *p-value* < 0.05 was considered statistically significant. SRS-2, Social Responsiveness Scale-Second Edition;.

TD, typically developing; ASD, autism spectrum disorder; SCI, Social Communication and Interaction; RRB, Restricted and Repetitive Behaviors; SD, standard deviation; η^2^, eta squared; BH, Benjamini-Hochberg; FDR, false discovery rate.

Hierarchical multiple regression analyses were conducted within the ASD group to examine associations between parent-reported measures (SRS-2 or AQ-J subscales) and clinician-rated autism severity (ADOS-2 total score). In Model 1, subscale scores were entered simultaneously as predictors. In Model 2, sex (male = 1, female = 0) was added. This hierarchical structure was chosen first to examine the variance in clinician-rated autism severity accounted for by parent-reported subscale scores (Model 1) and then to determine whether sex explained additional variance beyond these predictors (Model 2). This ordering was selected because the primary objective was to evaluate whether sex contributed uniquely to clinician-rated autism severity after accounting for parent-reported symptom profiles. Non-standardized regression coefficients (B), standard errors (SE), standardized coefficients (β), t values, p values, and 95% confidence intervals (CI) were reported. The model fit indices (R^2^ and adjusted R^2^) were calculated for each step.

To examine whether age was associated with discrepancies between parent-reported and clinician-rated autism severity, Spearman’s rank correlation analyses were conducted separately for males and females in the ASD group. Discrepancy scores were calculated as the difference between the standardized parent-reported and clinician-rated total scores (SRS-2 total score minus ADOS-2 total score; AQ-J total score minus ADOS-2 total score). Spearman’s correlation coefficient (ρ) was used because discrepancy scores were not assumed to be normally distributed. Statistical significance was defined as p < 0.05 (two-tailed).

## 3 Results

### 3.1 Participant characteristics

The participant demographics and cognitive characteristics are presented in **Table 2**. Sex distribution did not differ significantly between the ASD and TD groups (p = 0.423). Age did not differ significantly among the four groups (p = 0.469). Furthermore, no significant differences were observed among the groups in DNA-CAS total scores (p = 0.129). Overall, the TD and ASD groups were comparable with respect to age, sex distribution, and DN-CAS scores. Psychiatric comorbidities within the ASD group are summarized in **Supplementary Table 2**. Medication categories at the time of assessment are summarized in **Supplementary Table 3**.

**Table 2:** Participant demographics and cognitive characteristics.

Variables	TD				ASD					
	Male		Female		Male		Female			
	Mean	SD	Mean	SD	Mean	SD	Mean	SD	*χ^2^ or F* value	*p* value
Sex	N = 23		N = 16		N = 20		N = 20		0.641	0.423
Age	10.87	2.28	12.00	2.42	10.95	2.37	11.30	2.34	0.854	0.469
DN-CAS Total score	93.48	13.82	104.88	16.22	96.30	13.65	99.25	16.49	1.951	0.129

Values are presented as means and standard deviations (SD) unless otherwise indicated. Participants were categorized into typically developing (TD) and autism spectrum disorder (ASD) groups and further stratified by sex (male/female). Cognitive function was assessed using the Das–Naglieri Cognitive Assessment System (DN-CAS). Group differences in categorical variables (sex distribution) were examined using the chi-square (χ^2^) test. Continuous variables were analyzed using one-way analysis of variance (ANOVA) across the four groups (TD male, TD female, ASD male, ASD female). TD, typically developing; ASD, autism spectrum disorder; DN-CAS, Das–Naglieri Cognitive Assessment System; SD, standard deviation; χ^2^, chi-square statistic; ANOVA, analysis of variance.

### 3.2 Diagnostic effects on parent-reported measures and sex differences in clinician-rated autism severity

Two-way ANOVA was conducted to examine the effects of the diagnostic group (TD vs. ASD) and sex (male vs. female) on SRS-2 scores (**Table 1**). For the total score, a significant main effect of diagnosis was observed (p < 0.001), indicating higher parent-reported social responsiveness scores in the ASD group than in the TD group. Significant main effects of diagnosis were consistently found across all SRS-2 subscales, including Social Awareness, Social Cognition, Social Communication, Social Motivation, and Restricted Interests and Repetitive Behavior (all p < 0.001). Similar diagnostic effects were observed in the DSM-5–aligned indices, SCI and RRB (both p < 0.001). In contrast, no significant main effects of sex were detected on the total score or any subscale (all p > 0.05). No significant interactions between diagnosis and sex were observed for any of the SRS-2 measures (all p > 0.05), indicating that the magnitude of diagnostic differences did not differ according to sex.

A separate two-way ANOVA was conducted to examine AQ-J scores (**Supplementary Table 1**). A significant main effect of diagnosis was observed for the total score (p < 0.001), with higher parent-reported autistic trait scores in the ASD group. Diagnostic effects were also significant across all AQ-J subscales after FDR correction, including Social Skill, Attention Switching, Local Details, Communication, and Imagination (all p < 0.01). With respect to sex, no significant main effects were observed on the total score (p = 0.062) or on most subscales (all p > 0.05). However, a significant main effect of sex was found for Imagination (p = 0.003), which remained significant after FDR correction, with males scoring higher than females across the diagnostic groups. No significant interactions between diagnosis and sex were detected for any of the AQ-J measurements (all p > 0.05).

Within the ASD group, sex differences in clinician-rated autism severity were examined using ADOS-2 total and domain scores (**Table 3**). Males showed significantly higher ADOS-2 total scores than females (p = 0.034). No significant sex differences were observed for Reciprocal Social Interaction (p = 0.164), Social Affect (p = 0.102), Restricted and Repetitive Stereotyped Behaviors and Interests (p = 0.054), or Language and Communication (p = 0.126).

**Table 3:** Sex differences in ADOS-2 total and domain scores among participants with ASD.

Variables	ASD						
	Male		Female				
	Mean	SD	Mean	SD	*t* value	*p* value	Cohen’s d
Total score	12.50	2.74	10.75	2.27	2.199	**0.034**	0.695
Language and communication	3.60	0.99	3.15	0.81	1.567	0.126	0.495
Reciprocal social interaction	8.25	1.89	7.45	1.67	1.419	0.164	0.449
Social affect total	11.85	2.43	10.60	2.28	1.676	0.102	0.530
Restricted and repetitive stereotyped behaviors and interests	0.65	1.04	0.15	0.37	2.028	0.054	0.641

Values are presented as mean and standard deviation (SD). The severity of autism symptoms was assessed using the Autism Diagnostic Observation Schedule (Second Edition (ADOS-2). Male and female participants with autism spectrum disorder (ASD) were compared using independent-sample *t*-tests. Effect sizes are reported as Cohen’s d, representing the standardized mean difference in ADOS-2 scores between males and females. Bold values indicate statistical significance at *p* < 0.05. A two-tailed *p-value* < 0.05 was considered statistically significant. ADOS-2, Autism Diagnostic Observation Schedule, Second Edition; ASD, autism spectrum disorder; SD, standard deviation; η^2^, eta squared.

Taken together, diagnostic status robustly differentiated the TD and ASD groups in both parent-reported measures (SRS-2 and AQ-J). In contrast, sex differences were limited in the parent-reported measures and were also limited in the clinician-rated assessments, with a significant effect observed only for the ADOS-2 total score within the ASD group.

### 3.3 Associations between parent-reported social responsiveness and clinician-rated autism severity

Hierarchical multiple regression analyses were conducted within the ASD group to examine associations between SRS-2 subscale scores and ADOS-2 total scores (**Table 4**). In Model 1, the five SRS-2 subscales were entered simultaneously as predictors. This model was not statistically significant (R^2^ = 0.146, p = 0.346), and none of the SRS-2 subscales significantly predicted ADOS-2 total scores (all p > 0.05). In Model 2, sex was added as an additional predictor; this model was statistically significant (R^2^ = 0.331, p = 0.029), and sex was a significant independent predictor of ADOS-2 total scores (p = 0.005), indicating higher clinician-rated severity in males. None of the SRS-2 subscales reached statistical significance in this model.

**Table 4:** Hierarchical multiple regression analyses examining the association between SRS-2 subscales and ADOS-2 total scores among participants with ASD.

Dependent variable and covariate						95% confidence interval				
	B	SE	β	*t*-value	*p*-value	Lower	Upper	R^2^	Adjusted R^2^	*p*-value
ADOS-2 total score (Model 1)^1^								0.146	0.021	0.346
Social awareness	−0.047	0.043	−0.246	−1.098	0.280	−0.133	0.040			
Social cognition	−0.061	0.054	−0.319	−1.132	0.266	−0.170	0.048			
Social communication	0.007	0.058	0.029	0.115	0.909	−0.112	0.125			
Social motivation	−0.004	0.040	−0.019	−0.097	0.923	−0.085	0.077			
Repetitive behaviors and restricted interests	0.040	0.041	0.233	0.985	0.332	−0.043	0.124			
ADOS-2 total score (Model 2)'^2^								**0.331**	**0.210**	**0.029**
Social awareness	−0.078	0.040	−0.409	−1.967	0.058	−0.159	0.003			
Social cognition	−0.063	0.048	−0.331	−1.307	0.200	−0.161	0.035			
Social communication	0.049	0.054	0.217	0.912	0.369	−0.061	0.159			
Social motivation	−0.038	0.037	−0.184	−1.015	0.318	−0.114	0.038			
Repetitive behaviors and restricted interest	0.051	0.037	0.293	1.371	0.180	−0.025	0.126			
Sex	**2.441**	**0.808**	**0.469**	**3.021**	**0.005**	**0.797**	**4.085**			

Hierarchical multiple regression analyses were conducted within the autism spectrum disorder (ASD) group to examine the association between parent-reported social impairments assessed using the Social Responsiveness Scale–Second Edition (SRS-2) and clinician-rated autism symptom severity assessed using the Autism Diagnostic Observation Schedule–Second Edition (ADOS-2). In Model 1 (ADOS2 total1), the five SRS-2 treatment subscales (Social Awareness, Social Cognition, Social Communication, Social Motivation, and Restricted Interests and Repetitive Behaviors) were simultaneously entered as predictors. In Model 2 (ADOS2 total2), sex (male = 1, female = 0) was additionally included to evaluate whether sex independently contributed to ADOS-2 total scores beyond parent-reported social characteristics.

Unstandardized regression coefficients (B), standard errors (SE), standardized coefficients (β), t values, p values, and 95% confidence intervals (CI) are presented for each predictor. Model fit indices, including R^2^, adjusted R^2^, and model-level *p*-values, are shown for each step. Bold values indicate statistical significance at *p* < 0.05. A two-tailed *p-value* < 0.05 was considered statistically significant. SRS-2, Social Responsiveness Scale, Second Edition; ADOS-2, Autism Diagnostic Observation Schedule, Second Edition; SE, standard error; CI, confidence interval; ASD, autism spectrum disorder.

To further examine the association between these measures, complementary analyses were conducted using ADOS-2 domain scores as predictors of SRS-2 total scores (**Supplementary Table 4**). No robust associations were observed, and the results remained largely unchanged after adjustment for sex.

Overall, parent-reported social responsiveness strongly differentiated the diagnostic groups, whereas the correspondence between SRS-2 subscales and clinician-rated autism severity within the ASD group was limited. Sex was a significant independent predictor of ADOS-2 severity, and no SRS-2 subscale reached statistical significance after adjustment for sex.

### 3.4 Associations between parent-reported autistic traits and clinician-rated autism severity

Hierarchical multiple regression analyses were conducted within the ASD group to examine associations between AQ-J subscale scores and ADOS-2 total scores (**Supplementary Table 5**). In Model 1, the five AQ-J subscales were entered simultaneously as predictors. The overall model was not statistically significant (R^2^ = 0.185, p = 0.202), and none of the AQ-J subscales significantly predicted ADOS-2 total scores (all p > 0.05). Sex was added as an additional predictor in Model 2, and the overall model was statistically significant (R^2^ = 0.317, p = 0.025). In this model, sex was a significant independent predictor (p = 0.009), whereas none of the AQ-J subscales were statistically significant. Complementary analyses using ADOS-2 symptom domains as predictors of AQ-J total scores (**Supplementary Table 6**) did not reveal robust associations.

Overall, associations between parent-reported autistic traits (AQ-J) and clinician-rated autism severity were limited. Although none of the AQ-J subscales significantly predicted ADOS-2 severity, sex was a significant independent predictor in Model 2. In contrast, no robust domain-level predictors of AQ-J total scores were identified.

### 3.5 Age and parent–clinician discrepancy

Spearman’s rank correlation analyses were conducted separately for males and females within the ASD group to examine associations between age and parent–clinician discrepancy scores (**Table 5**). Among males, no significant associations were observed between age and discrepancy scores for either the SRS-2 total score minus ADOS-2 total score (ρ = −0.025, p > 0.05) or the AQ-J total score minus ADOS-2 total score (ρ = 0.161, p > 0.05). Among females, age was significantly negatively correlated with the SRS-2–ADOS-2 discrepancy score (ρ = −0.484, p < 0.05), indicating that older age was associated with a smaller discrepancy between parent-reported and clinician-rated autism severity. No significant association was observed between age and the AQ-J–ADOS-2 discrepancy score in females (ρ = 0.023, p > 0.05).

**Table 5:** Correlations between age and parent–clinician discrepancy scores with ASD, stratified according to sex.

Sex	SRS-2 total score - ADOS-2 total score	AQ-J total score – ADOS-2 total score
Male	−0.025	0.161
Female	−0.484*	0.023

Spearman’s rank correlation analyses were conducted separately for males and females to examine the association between age and the discrepancy between parent-reported measures and clinician-rated autism severity. Discrepancy scores were calculated as the difference between standardized parent-reported and clinician-rated scores (SRS-2 total score – ADOS-2 total score; AQ-J total score – ADOS-2 total score). Spearman’s correlation coefficients (ρ) represent associations between age and discrepancy scores within each sex group. A negative correlation indicated that older age was associated with a smaller parent–clinician discrepancy. Bold values indicate statistical significance at p < 0.05. *p < 0.05 (two-tailed). SRS-2, Social Responsiveness Scale, Second Edition; ADOS-2, Autism Diagnostic Observation Schedule, Second Edition; AQ-J, Autism-Spectrum Quotient, Japanese version.

## 4 Discussion

Overall, this study examined discrepancies between parent-reported and clinician-rated measures of autism severity within an informant discrepancy framework. Parent-reported measures robustly differentiated ASD and typically developing groups but showed limited associations with clinician-rated ADOS-2 severity within the ASD group. Sex-related differences were observed in the ADOS-2 total scores, but were limited in parent-reported measures. Parent-reported instruments were strongly correlated with one another but only weakly associated with ADOS-2 severity. Further, age was associated with a reduced discrepancy between SRS-2 and ADOS-2 scores among females. These findings suggest that parent-reported and clinician-rated measures capture overlapping but distinct aspects of autism symptom expression. The ADOS-2 captured sex-related differences in clinician-rated severity that were not apparent in parent-reported assessments, though these differences require cautious interpretation in light of documented instrument limitations.

### 4.1 Cross-method differences between parent-reported and clinician-rated measures

Parent-reported measures (SRS-2 and AQ-J) were strongly interrelated, but their association with clinician-rated ADOS-2 severity was weak. This pattern suggests that parent questionnaires and structured observational assessments capture different aspects of autism-related behavior shaped by the assessment context. Parent reports reflect behaviors across naturalistic, longitudinal settings, whereas the ADOS-2 evaluates behavior within a standardized, time-limited interaction designed to elicit core social-communication features (12, 32). Strong correlations between parent-reported instruments may therefore reflect shared method variance and common contextual framing (33), whereas weaker associations with the ADOS-2 likely reflect cross-method differences rather than inconsistency. Previous studies have consistently documented modest associations between questionnaire-based ratings and observational diagnostic measures (34, 35). Parent-reported instruments may also be influenced by broader behavioral and developmental characteristics that influence the global impressions of functioning. The specificity of parent-reported screeners varies according to child characteristics and co-occurring behaviors (36, 37). In contrast, ADOS-2 was explicitly designed to standardize the elicitation and scoring of observable autism-related behaviors (12, 32), thereby providing a standardized context for observing and scoring autism-related behaviors. From this perspective, a lower parent–clinician correspondence may reflect differences between behaviors observed in everyday contexts and those observed during structured clinical assessment.

Differences between the SRS-2 and AQ-J may help explain the variation in age-related patterns observed across measures. The SRS-2 primarily indexes the severity of social impairment and everyday functional impact, whereas the AQ-J was originally developed as a dimensional measure of autistic traits. Consequently, the SRS-2 may be more sensitive to age-related variation in social functioning and parent–clinician correspondence. In contrast, trait-oriented measures such as the AQ-J may show weaker alignment with clinician-rated severity across age groups. Contemporary informant discrepancy models emphasize that cross-rater differences are often meaningful and context-dependent, rather than measurement errors (7, 9, 31). Informants may access different behavioral demands and environments, and each assessment modality may index distinct dimensions of symptom expression. Therefore, considering these discrepancies as informative may enhance the interpretation of autism severity across different settings.

### 4.2 Sex differences emerging in clinician-rated assessment

Sex differences were evident in clinician-rated ADOS-2 scores, but were limited in parent-reported measures. One possible explanation is that structured observational assessments and parent-reported measures may capture different aspects of autism-related behavior across assessment contexts. The ADOS-2 standardizes behavioral elicitation and scoring (12, 32), providing a standardized context for observing and scoring autism-related behaviors. Consequently, behaviors observed during structured assessments may differ from those reported by parents across everyday settings. In contrast, parent-reported instruments aggregate behaviors across naturalistic settings and may reflect broader functional adaptations rather than behaviors observed during structured assessment procedures. Previous studies have reported inconsistent sex effects in questionnaire-based measures, whereas clinician-administered tools have demonstrated clear differences (21, 22, 38). This pattern suggests that assessment context may influence how sex-related differences are expressed and identified. Taken together, these findings suggest that structured observational assessments and parent-reported measures may capture different aspects of autism-related behavior. Structured assessments provide information about behaviors observed under standardized conditions, whereas parent reports may capture functioning across a broader range of everyday contexts.

However, an alternative interpretation warrants consideration. The ADOS-2 was developed and normed predominantly in male samples, and accumulating evidence demonstrates its limited sensitivity for detecting autism in females (5, 20). Males scoring higher on the ADOS-2 may therefore partly reflect instrument-level bias rather than genuine differences in symptom severity, and the finding that sex significantly predicted ADOS-2 scores in the regression analyses (Model 2) may be consistent with this known measurement bias. Furthermore, autistic females may engage in camouflaging or masking—the deliberate or habitual adoption of neurotypical social strategies to compensate for autistic traits—which can suppress directly observable behaviors during structured clinical assessments while behaviors in naturalistic settings (captured by parent reports) remain relatively unaffected (27). These possibilities suggest that parent-reported measures may provide information about everyday behavioral functioning in autistic females that is not fully captured by clinician-rated structured observations.

### 4.3 Developmental reduction in parent–clinician discrepancy

Age was associated with a reduced discrepancy between SRS-2 and ADOS-2 scores, indicating greater parent–clinician agreement among older female participants. One possible interpretation, among several competing explanations, is that older participants may show greater behavioral similarity across assessment contexts, which could contribute to increased agreement between parental observations and structured clinical assessments. However, given the cross-sectional design of the present study, the observed age differences reflect between-individual variation rather than within-individual longitudinal change, and developmental interpretations should be made with caution (9, 12). Informant discrepancy research further indicates that cross-rater agreement tends to increase when behaviors are more clearly defined and less context-dependent (37, 38).

An alternative explanation is provided by the camouflaging literature. The age-related reduction in discrepancy among females may not reflect greater agreement in symptom expression across contexts, but instead age-related differences in camouflaging behaviors (27). Older girls may be more adept at masking autistic behaviors during structured clinical assessments while continuing to display these behaviors in everyday contexts observed by parents, producing apparent convergence between parent reports and clinician observations without reflecting true agreement across informants. This interpretation is consistent with evidence that camouflaging is more prevalent and more frequently reported in autistic females than males, and may have important clinical implications for the interpretation of reduced discrepancy in older girls.

Additionally, this age-related reduction in discrepancy was observed for the SRS-2 but not for the AQ-J discrepancy scores. The SRS-2 primarily indexes the severity of social impairment and everyday functional impact, which may align more closely with clinician-rated severity among older participants. In contrast, the AQ-J was developed as a dimensional trait measure of autistic characteristics (14), and therefore, it may be less sensitive to age-related variation in observable symptom severity. These findings suggest that age-related differences in informant discrepancy depend on the measurement focus of the instrument.

### 4.4 Limitations

This study had several limitations. First, the cross-sectional design precludes within-individual developmental conclusions; longitudinal studies are needed to determine whether the observed age-related differences in parent–clinician discrepancy reflect developmental changes within individuals. Second, parent-reported data relied on a single informant; including additional reporters (e.g., teachers) would enable a more comprehensive evaluation across contexts. Third, the sample size was modest, particularly for sex-stratified analyses within the ASD group, and warrants replication in larger cohorts. A *post-hoc* sensitivity analysis indicated that the study was powered to detect only relatively large effects (minimum detectable f^2^ = 0.304–0.410 across regression models; 80% power, α = 0.05); therefore, non-significant findings should be interpreted with appropriate caution. Fourth, although cognitive ability did not differ significantly among groups, other factors, such as adaptive functioning and comorbid symptoms, were not fully examined and may have influenced parent-reported ratings. Fifth, the age range studied (9–15 years) may overlap with developmental periods during which the presentation of autism-related behaviors varies across contexts, potentially influencing clinician-rated assessments.

Overall, this study examined discrepancies between parent-reported and clinician-rated measures of autism severity. Parent-reported instruments were strongly related to one another but showed limited correspondence with ADOS-2 severity. Sex differences were detected in clinician-rated assessments but not in parent reports, and age was associated with reduced discrepancy between SRS-2 and ADOS-2 scores, whereas AQ-J discrepancy scores were unrelated to age. These findings suggest that parent-reported and clinician-rated measures capture related but distinct dimensions of autism-related characteristics. Rather than indicating inconsistency, differences between informants may reflect the distinct contexts and perspectives represented by each assessment modality. Recognizing informant discrepancies as meaningful may improve the interpretation of autism-related characteristics in different assessment contexts and age groups.

## Data Availability

The raw data supporting the conclusions of this article will be made available by the authors, without undue reservation.
