# Supplementary material for: Discrepancies between parent-reported and clinician-rated autism severity: roles of sex and age
Source: Front Psychiatry. 2026 Aug 17;17:1823090. doi: 10.3389/fpsyt.2026.1823090 (PMC13528303; doi:10.3389/fpsyt.2026.1823090)
Supplement: Supplementary file 1 [file DataSheet1.pdf]

Supplementary Material

1 Supplementary Figures and Tables

1.1. Supplementary Figure

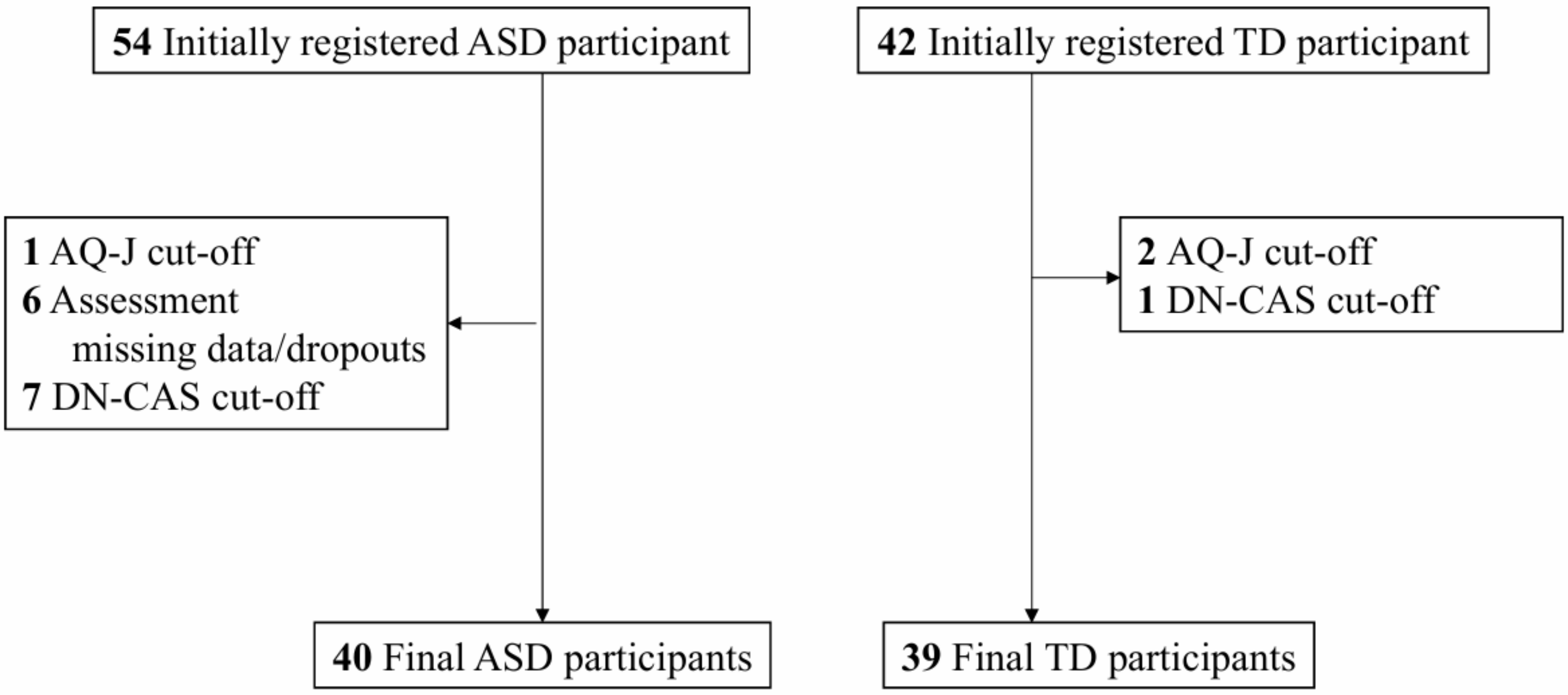

Supplementary Figure 1. Flow diagram of participant selection

Flow diagram illustrating participant enrollment and exclusion procedures. Among 54 initially registered participants with autism spectrum disorder (ASD), 1 was excluded based on the AQ-J cut-off, 6 were excluded due to missing assessment data or dropout, and 7 were excluded based on the DN-CAS cut-off. A total of 40 participants with ASD were included in the final analyses.

Among 42 initially registered typically developing (TD) participants, 2 were excluded based on the AQ cut-off, and 1 was excluded based on the DN-CAS cut-off, resulting in 39 TD participants in the final sample.

Abbreviations: ASD, autism spectrum disorder; TD, typically developing; AQ-J, Autism-Spectrum Quotient–Japanese version; DN-CAS, Das–Naglieri Cognitive Assessment System.

1.2. Supplementary Tables

Supplementary Table 1. Psychiatric comorbidities of the ASD sample

Comorbidities	N (%)
Attention-Deficit/Hyperactivity Disorder	5 (12.5)
Obsessive-Compulsive Disorder	1 (2.5)
Tic Disorders	1 (2.5)

Values are presented as the number of participants with each comorbid diagnosis and the corresponding percentage of the total ASD sample (N = 40). Percentages were calculated as (n / 40) × 100. Diagnoses were determined according to DSM-5-TR criteria.

Abbreviations: ASD, autism spectrum disorder; DSM-5-TR, Diagnostic and Statistical Manual of Mental Disorders, Fifth Edition, Text Revision.

Supplementary Table 2. Medication categories in the ASD sample

Medication categories	N (%)
Atypical antipsychotics	8 (20.0)
ADHD medications	6 (15.0)
Benzodiazepines	3 (7.5)
melatonin receptor agonists	3 (7.5)
Orexin receptor antagonists	2 (5.0)
Antidepressants	2 (5.0)
Non-benzodiazepine hypnotics	1 (2.5)
Mood stabilizers / antiepileptic drugs	1 (2.5)

Values are presented as the number of participants receiving each medication category and the corresponding percentage of the total ASD sample (N = 40). Percentages were calculated as (n / 40) × 100. Participants may have been prescribed more than one medication; therefore, the total percentage exceeds 100%. Medication categories were defined based on current prescriptions at the time of assessment.

Abbreviations: ASD, autism spectrum disorder; ADHD, attention-deficit/hyperactivity disorder.

Supplementary Table 3. Autism-spectrum quotient–Japanese version (AQ-J) scores according to diagnostic group and sex

Measure	TD		ASD		Sex	Diagnosis	Interaction
	Male Mean (SD)	Female Mean (SD)	Male Mean (SD)	Female Mean (SD)	(F, p, Partial η²)	(F, p, Partial η²)	(F, p, Partial η²)
Total score	14.74 (4.75)	11.38 (5.44)	29.00 (8.65)	26.55 (7.51)	$F_{1,75} = 3.585$ $p = 0.062$ Partial $\eta^2 = 0.046$	**$F_{1,75} = 91.897$ $p = 1.151\times10^{-14}$ Partial $\eta^2 = 0.551$**	$F_{1,75} = 0.089$ $p = 0.767$ Partial $\eta^2 = 0.001$
Social skill	2.83 (2.29)	2.75 (2.35)	7.05 (2.65)	6.40 (2.74)	$F_{1,75} = 0.405$ $p = 0.526$ Partial $\eta^2 = 0.005$	**$F_{1,75} = 47.620$ $p = 1.424\times10^{-9}$ Partial $\eta^2 = 0.388$**	$F_{1,75} = 0.253$ $p = 0.616$ Partial $\eta^2 = 0.003$
Attention switching	3.22 (1.51)	3.25 (2.02)	6.10 (2.17)	5.85 (2.23)	$F_{1,75} = 1.072$ $p = 0.304$ Partial $\eta^2 = 0.014$	**$F_{1,75} = 49.827$ $p = 7.202\times10^{-10}$ Partial $\eta^2 = 0.399$**	$F_{1,75} = 0.215$ $p = 0.645$ Partial $\eta^2 = 0.003$
Local details	3.35 (1.85)	2.95 (1.61)	4.45 (2.14)	4.80 (2.44)	$F_{1,75} = 0.069$ $p = 0.794$ Partial $\eta^2 = 0.001$	**$F_{1,75} = 7.614$ $p = 7.273\times10^{-3}$ Partial $\eta^2 = 0.092$**	$F_{1,75} = 0.217$ $p = 0.643$ Partial $\eta^2 = 0.003$
Communication	2.13 (1.55)	1.00 (1.32)	5.85 (2.81)	5.45 (2.06)	$F_{1,75} = 2.750$ $p = 0.101$ Partial $\eta^2 = 0.035$	**$F_{1,75} = 78.365$ $p = 2.842\times10^{-13}$ Partial $\eta^2 = 0.511$**	$F_{1,75} = 0.626$ $p = 0.431$ Partial $\eta^2 = 0.008$
Imagination	3.22 (1.98)	1.81 (1.76)	5.55 (2.21)	4.05 (2.33)	**$F_{1,75} = 9.360$ $p = 0.003$ Partial $\eta^2 = 0.111$**	**$F_{1,75} = 23.168$ $p = 7.512\times10^{-6}$ Partial $\eta^2 = 0.236$**	$F_{1,75} = 0.010$ $p = 0.920$ Partial $\eta^2 = 1.338\times10^{-4}$

Values are presented as mean and standard deviation (SD). Autistic traits were assessed using the Autism-Spectrum Quotient–Japanese version (AQ-J), which includes the total score and five subscales (Social skill, Attention switching, Local details, Communication, and Imagination). Two-way analysis of variance (ANOVA) was conducted for each AQ-J measure, with diagnostic group (typically developing [TD] vs. autism spectrum disorder [ASD]) and sex (male vs. female) as between-subject factors. F values, p values, and effect sizes are reported for the main effects of sex and diagnosis, as well as for their interaction.

Effect sizes are expressed as partial eta squared (partial η^2). Benjamini–Hochberg (BH) false discovery rate (FDR) correction was applied separately to the p-values for each effect type (sex, diagnosis, and interaction) across the eight AQ-J measures. Bold values indicate statistical significance after FDR correction. A two-tailed *p*-value < 0.05 was considered statistically significant.

Abbreviations: AQ-J, Autism-Spectrum Quotient, Japanese version; TD, typically developing; ASD, autism spectrum disorder; SD, standard deviation; ANOVA, analysis of variance; η^2, eta squared; BH, Benjamini–Hochberg; FDR, false discovery rate.

Supplementary Table 4. Hierarchical multiple regression analyses examining associations between ADOS-2 symptom domains and SRS-2 total scores with ASD

Dependent variable and covariate	B	SE	β	*t*-value	*p*-value	95% confidence interval		R^2	Adjusted R^2	*p*-value
						Lower	Upper			
SRS2 total[1]								0.131	0.059	0.162
Language and communication	1.202	2.496	0.087	0.482	0.633	-3.860	6.265			
Reciprocal and interaction	-2.798	1.292	-0.396	-2.165	0.037	-5.419	-0.177			
Restricted and repetitive stereotyped behaviors and interests	2.019	2.478	0.128	0.815	0.420	-3.006	7.045			
SRS2 total[2]								0.139	0.041	0.250
Language and communication	0.945	2.562	0.068	0.369	0.714	-4.256	6.145			
Reciprocal and interaction	-2.853	1.309	-0.403	-2.180	0.036	-5.510	-0.197			
Restricted and repetitive stereotyped behaviors and interests	1.575	2.624	0.100	0.600	0.552	-3.751	6.902			
Sex	2.420	4.310	0.096	0.562	0.578	-6.329	11.169			

Hierarchical multiple regression analyses were conducted within the autism spectrum disorder (ASD) group to examine the associations between the clinician-rated autism symptom domains assessed using the Autism Diagnostic Observation Schedule–Second Edition (ADOS-2) and parent-reported social impairment assessed using the Social Responsiveness Scale–Second Edition (SRS-2) total T-score. In Model 1 (SRS2 total1), three ADOS-2 domain scores—Language and Communication, Reciprocal Social Interaction, and Restricted and Repetitive Stereotyped Behaviors and Interests—were simultaneously entered as predictors. In Model 2 (SRS2 total2), sex (male = 1, female = 0) was additionally included to assess whether sex independently contributed to SRS-2 total scores beyond the clinician-rated symptom severity. Unstandardized regression coefficients (B), standard errors (SE), standardized coefficients (β), t values, p values, and 95%

confidence intervals (CI) are reported for each predictor. Model fit indices (R^2, adjusted R^2, and overall model p values) are presented for each step. Bold values indicate statistical significance at $p < 0.05$. A two-tailed *p*-value < 0.05 was considered statistically significant.

Abbreviations: ADOS-2, Autism Diagnostic Observation Schedule, Second Edition; SRS-2, Social Responsiveness Scale, Second Edition; ASD, autism spectrum disorder; SE, standard error; CI, confidence interval; R^2, coefficient of determination; β, standardized regression coefficient.

Supplementary Table 5. Hierarchical multiple regression analyses examining associations between parent-reported autistic traits (AQ-J) and ADOS-2 total scores with ASD

Dependent variable and covariate	B	SE	β	*t*-value	*p*-value	95% confidence interval		R^2	Adjusted R^2	*p*-value
						Lower	Upper			
ADOS2 total[1]								0.185	0.065	0.202
Social skill	0.101	0.177	0.103	0.571	0.572	-0.259	0.462			
Attention switching	-0.423	0.258	-0.349	-1.638	0.111	-0.948	0.102			
Local details	0.290	0.188	0.250	1.542	0.132	-0.092	0.672			
Communication	-0.099	0.226	-0.091	-0.436	0.666	-0.559	0.361			
Imagination	0.008	0.203	0.007	0.039	0.969	-0.404	0.420			
ADOS2 total[2]								**0.340**	**0.220**	**0.025**
Social skill	0.072	0.162	0.074	0.446	0.659	-0.258	0.403			
Attention switching	-0.354	0.237	-0.292	-1.493	0.145	-0.837	0.129			
Local details	0.326	0.172	0.281	1.894	0.067	-0.024	0.677			
Communication	-0.103	0.207	-0.096	-0.499	0.621	-0.524	0.317			
Imagination	-0.160	0.195	-0.144	-0.821	0.418	-0.557	0.237			
Sex	**2.187**	**0.787**	**0.420**	**2.780**	**0.009**	**0.587**	**3.787**			

Hierarchical multiple regression analyses were conducted within the autism spectrum disorder (ASD) group to examine the associations between parent-reported autistic traits assessed by the Autism-Spectrum Quotient–Japanese version (AQ-J) and clinician-rated autism symptom severity assessed using the Autism Diagnostic Observation Schedule–Second Edition (ADOS-2). In Model 1 (ADOS2 total1), the five AQ-J subscales (Social Skill, Attention Switching, Local Details, Communication, and Imagination) were simultaneously entered as predictors. In Model 2 (ADOS2 total2), sex (male = 1, female = 0) was

additionally included to evaluate whether sex independently contributed to ADOS-2 total scores beyond parent-reported autistic traits. Non-standardized regression coefficients (B), standard errors (SE), standardized coefficients (β), t values, p values, and 95% confidence intervals (CI) are reported for each predictor. Model fit indices, including R^2, adjusted R^2, and model-level *p-values,* are shown for each step. Bold values indicate statistical significance at $p < 0.05$. A two-tailed p-value < 0.05 was considered statistically significant.

Supplementary Table 6. Hierarchical multiple regression analyses examining associations between ADOS-2 symptom domains and AQ-J total scores with ASD

Dependent variable and covariate	B	SE	β	*t*-value	*p*-value	95% confidence interval		R^2	Adjusted R^2	*p*-value
						Lower	Upper			
AQ-J total[1]								0.126	0.053	0.178
Language and communication	1.829	1.587	0.209	1.153	0.257	-1.389	5.047			
Reciprocal and interaction	-1.782	0.822	-0.398	-2.169	0.037	-3.449	-0.116			
Restricted and repetitive stereotyped behaviors and interests	1.592	1.575	0.159	1.010	0.319	-1.603	4.786			
AQ-J total[2]								0.150	0.053	0.212
Language and communication	1.544	1.613	0.177	0.957	0.345	-1.731	4.819			
Reciprocal and interaction	-1.844	0.824	-0.411	-2.237	0.032	-3.517	-0.171			
Restricted and repetitive stereotyped behaviors and interests	1.099	1.652	0.110	0.665	0.510	-2.255	4.454			
Sex	2.681	2.714	0.168	0.988	0.330	-2.829	8.191			

Hierarchical multiple regression analyses examined the associations between clinician-rated autism symptom domains assessed by the Autism Diagnostic Observation Schedule–Second Edition (ADOS-2) and parent-reported autistic traits assessed by the Autism-Spectrum Quotient–Japanese version (AQ-J) total score within the autism spectrum disorder (ASD) group. In Model 1 (AQ-J total1), three ADOS-2 domain scores—Language and Communication, Reciprocal Social Interaction, and Restricted and Repetitive Stereotyped Behaviors and Interests—were simultaneously entered as predictors of AQ-J total scores. In Model 2 (AQ-J total2), sex (male = 1, female = 0) was additionally included to examine whether sex independently contributed to parent-reported autistic traits beyond clinician-rated symptom severity. Non-standardized regression coefficients (B), standard errors (SE), standardized coefficients (β), t values, p values,

and 95% confidence intervals (CI) are reported for each predictor. Model fit indices (R^2, adjusted R^2, and overall model p values) are presented for each step. Bold values indicate statistical significance at $p < 0.05$. A two-tailed *p-value* < 0.05 was considered statistically significant.

Abbreviations: ADOS-2, Autism Diagnostic Observation Schedule, Second Edition; AQ-J, Autism-Spectrum Quotient, Japanese version; ASD, autism spectrum disorder; SE, standard error; CI, confidence interval; R^2, coefficient of determination; β, standardized regression coefficient.
